# Decreased Expression of Ileal Thyroid Hormone Transporters in a Hypothyroid Patient: A Case Report

**DOI:** 10.3389/fendo.2021.664839

**Published:** 2021-05-26

**Authors:** Chae Won Chung, Eun Young Mo, Gyung Seo Jung, Yoo Hyung Kim, Sun Wook Cho, Do Joon Park, Jeong Mo Bae, Young Joo Park

**Affiliations:** ^1^ Department of Internal Medicine, Seoul National University Hospital, Seoul, South Korea; ^2^ Department of Internal Medicine, The Catholic University of Korea Incheon St. Mary’s Hospital, Incheon, South Korea; ^3^ Department of Internal Medicine, Seoul National University College of Medicine, Seoul, South Korea; ^4^ Department of Pathology, Seoul National University Hospital, Seoul, South Korea; ^5^ Department of Molecular Medicine and Biopharmaceutical Sciences, Graduate School of Convergence Science and Technology, Seoul National University, Seoul, South Korea

**Keywords:** hypothyroidism, thyroid hormone transporter, malabsorption of levothyroxine, immunohistochemistry, levothyroxine absorption test

## Abstract

**Background:**

Malabsorption of levothyroxine (LT4) is a common problem faced in clinical practice. It is usually solved, if there are no complexities including gastrointestinal absorption disorder, by taking medicines on an empty stomach and avoiding foods interfering with LT4. Herein we present a rare case of a patient exhibiting malabsorption of LT4 with decreased membranous expression of ileal transporters.

**Case:**

The 22-Year-old female presented with sustained hypothyroid status despite medication of 7.8 μg/kg LT4. Medical history and LT4 absorption test (the absorption rate 8.4%) excluded pseudomalabsorption. No organic gastrointestinal disorder was found in the patient by blood chemistry, endoscopies, and abdominal computed tomography scan. The immunohistochemical analysis showed decreased membranous expression of LAT1 and LAT2 in distal ileum and ascending colon in the patient compared to 20 controls who have no thyroid disease. The expression of MCT8 in colon appeared at both nucleus and brush border in the patient, while it was limited to brush border in controls. The expression of other transporters was similar between the patient and controls.

**Conclusion:**

The changes of the expression of LAT1 and LAT2 in this patient showing LT4 malabsorption might help to understand the role of intestinal transporters in the absorption of LT4 in humans. The functional relevance of the decrement of LAT1 and LAT2 in this patient remains to be elucidated.

## Introduction

Hypothyroidism is one of the most common endocrinologic disorders with a prevalence ranging from 0.5% to 4-5% (1–3). Thyroid hormone deficiency is treated with oral supplement of synthetic thyroxine (levothyroxine, LT4). The usual daily dose of LT4 is 1.6 µg/kg, and it is adjusted by body weight, age, and the degree of hypothyroidism (4–6). However, despite aberrantly higher LT4 doses, some patients retain their hypothyroid status. There are several causes reducing intestinal absorption of LT4, including organic diseases of the gastrointestinal tract (e.g. inflammatory bowel disease, celiac disease, cystic fibrosis, and jejuno-ileal bypass surgery), pharmacodynamic interactions with medications or food (e.g. antacids, calcium, oral iron supplementation, statin, grapefruit juice, soy products, and coffee), and pregnancy (7–10). The other cause is nonadherence to LT4 replacement, namely pseudomalabsorption (11), and this has been proposed as a main cause (4, 12). This factitious malabsorption is distinguished by LT4 absorption test, which measures serum level of free T4 after loading high oral dose of LT4 (13, 14). However, there exist few cases showing true malabsorption without any causes (15–17).

As one of the possible causes of true malabsorption, interrupted function or expression of ileal LT4 transporters has been proposed (18). Since the first report of Allan-Herndon-Dudley syndrome and discovery of culprit mutation of monocarboxylate transporter 8 (MCT8) gene (19), which causes psychomotor retardation by affecting thyroid hormone uptake in neurons, the importance of LT4 transporter was highlighted. Over the last two decades, clinical studies and experimental evidence have shown LT4 transporters including organic anion transporting polypeptide (OATP) family (20, 21), the Na-taurocholate co-transporting polypeptide (NTCP) (22), the L-type amino acid transporter 1 and 2 (LAT1 and LAT2) (23, 24), MCT8 (25) and MCT10 (26).

However, experiments are rare in which cells are directly collected from humans to confirm the expression of transporters (27) – most results are derived from rodents or cell-lines transfected with human complementary DNA.

In this study, we aimed to investigate the expressions of ileal transports in a patient who presented a marked malabsorption of LT4 without gastrointestinal malabsorptive disorder comparing with controls.

## Case Report

**Figure 1:**
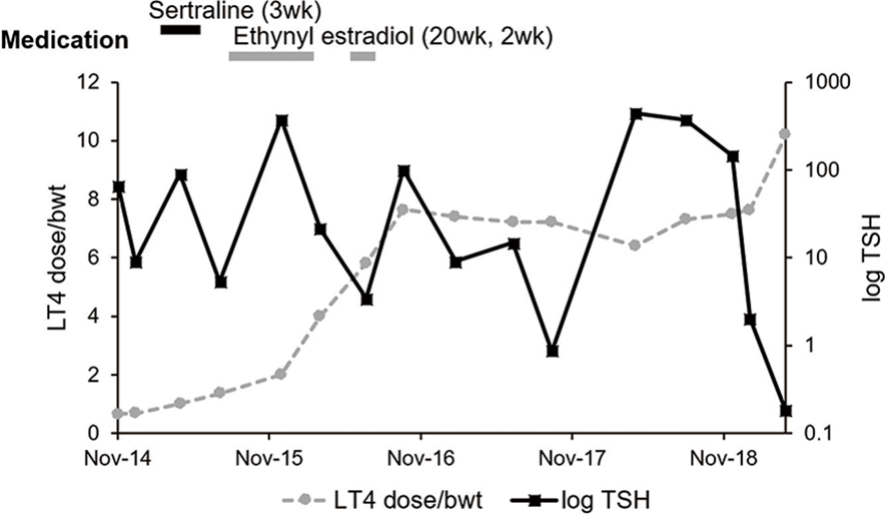
Medical history, log TSH, and LT4 dose per bodyweight of the case patient. The dose of sertraline and ethynyl estradiol were 50 mg per day and 0.03 mg per day, respectively. The beginning dates of each medication were May 2015, September 2015, and September 2016. There was fluctuation of serum TSH level in spite of increasing LT4 dose over bodyweight. LT4, levothyroxine; Bwt, bodyweight.

In 2014, an 18-year-old female visited Incheon St. Mary’s Hospital (A) because of fatigue. She was diagnosed with hypothyroidism from Hashimoto’s thyroiditis, and her serum TSH was 66.18 mIU/L, and free T4 was 0.51 ng/dL. Based on her body weight of 75 kg, 75 μg/day (1 μg/kg/day) of LT4 was started, but TSH was persistently high and amenorrhea occurred. The LT4 dose was gradually increased and finally reached 800 μg/day, but her TSH level failed to be normalized (**Figure 1**). Despite a combination of liothyronine and LT4 with corresponding dose of 300 μg/day of LT4, her TSH and free T4 were also not normalized; TSH level was 369.45 mIU/L, and free T4 was 0.2 ng/dL.

Under hypothyroid status, the patient was diagnosed with polycystic ovarian syndrome (PCOS) and took estradiol and progesterone for a short period and very irregularly. However, before and after administration of hormone therapy, the patient retained her hypothyroid status, which could make the effect of estrogen on LT4 malabsorption excluded. Her body weight change (**Figure 1**) also could not explain the aberrantly high LT4 dose. Except PCOS, there was no past medical, family, and psychosocial history correlated with thyroid function and gastrointestinal absorption disorder. She had no other relevant previous intervention than LT4 administration for hypothyroidism.

In 2018, the patient was referred to Seoul National University Hospital (B). She was taking 600 μg/day of LT4, her body weight was 80.0 kg. Her TSH was 29.85 mIU/L, and free T4 was 0.7 ng/dL. She denied pregnancy and gastrointestinal symptoms involving dysphagia, abdominal discomfort, vomiting, and diarrhea. She also had no symptoms related with lactose intolerance. Laboratory screening tests showed normal range; hemoglobin 12.1 g/dL (12-16 g/dL), albumin 4.7 g/dL (3.3-5.2 g/dL), calcium 9.8 mg/dL (8.8-10.5 mg/dL), phosphorus 4.4 mg/dL (2.5-4.5 mg/dL), CRP 0.14 mg/dL (0-0.5 mg/dL), WBC 10,540/μL (4,000-10,000/μL), and platelet 315,000/μL (130,000-400,000/μL). Liver and renal function were intact: aspartate transaminase 29 IU/L (1-40 IU/L), alanine transferase 43 IU/L, PT INR 0.90 (0.8-1.2), BUN 14 mg/dL (10-26 mg/dL), creatinine 0.97 mg/dL (0.7-1.4 mg/dL), and MDRD eGFR 71.5 mL/min/1.73. Gastroduodenoscopy and colonoscopy with biopsy ruled out inflammatory bowel disease, *Helicobacter pylori* infection, and celiac disease. Capsule endoscopy and abdominal computed tomography (CT) showed nonspecific mild small bowel thickening which was confirmed by an enterologist as less possibility of organic gastrointestinal malabsorptive disorder. All these results supported that there was low possibility of organic gastrointestinal absorptive disorder. Both lung area covered in abdominal CT also showed normal presentation and cystic fibrosis was excluded.

LT4 absorption test was done by one day-administration of 1,000 μg of LT4 and absorption rate was calculated by the equation using total T4, free T4, and volume of distribution (13). After loading LT4 1000 μg at Day 1, TSH and total T4 were measured at baseline, 1 hour, and 2 hours after LT4 ingesting. The equation used for LT4 absorption rate was the following;

LT4 absorption [%]=[(peak Δtotal T4 (μg/dL)×Vd (dL)÷administered dose of LT4 (μg)× 100](Vd=volume of distribution, 0.442×body mass index(BMI))

**Figure 2:**
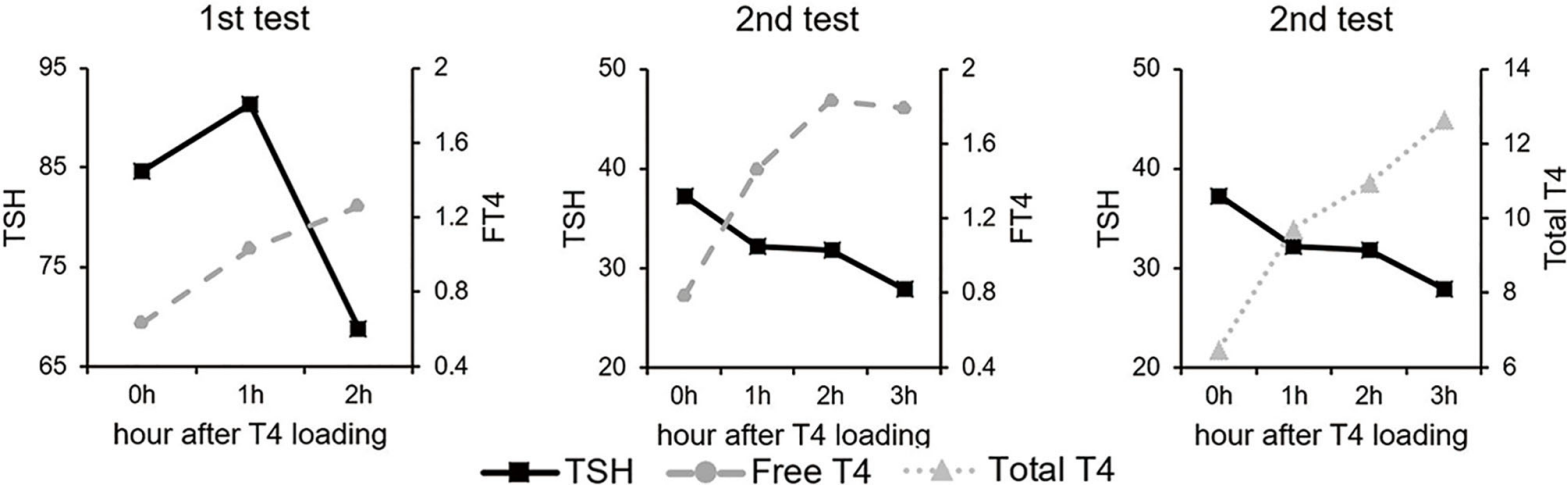
LT4 absorption test. The serum total T4 level increased and TSH level decreased in response to high dose of LT4 ingestion. Absorption rate of LT4 was 8.4%.

Because total T4 was not checked after loading of LT4 1,000 μg, re-loading of LT4 was done after 2 days. The test showed the absorption rate of 8.4% (**Figure 2**), and true malabsorption status of LT4 without obvious organic problems was confirmed.

To analyze ileal thyroid hormone transporters’ expression, the ileal sample of the patient was obtained from her terminal ileum and ascending colon. For control tissues, formalin-fixed paraffin embedded (FFPE) samples of 63 consecutive subjects aged 50 years or less were selected by retrospective review of colon cancer patients who underwent total colectomy from 2010 to 2012 at Seoul National University Hospital. Finally, 20 FFPE pairs of terminal ileum and ascending colon were obtained after exclusion of previous history of thyroid diseases, any medication including antibiotics or probiotics within 4 weeks before biopsy or surgery (n=32), gastrointestinal absorption disorder or abdominal surgery (n=7), or unavailable FFPEs (n=4). Controls consisted of 14 men and 6 women, and the mean age was 41 ± 6 years. For this retrospective review of FFPE, the study was approved by the Institutional Review Board of Seoul National University Hospital (No. H-1506-047-679). The case patient signed her informed consent, and the consent for controls were waived according to the approved protocol by the ethics committee of the institution and based on Helsinki declaration.

The 2mm-sized core was obtained in each block of terminal ileum and ascending colon of controls after confirming normal histology by one pathologist, and was subjected to tissue microarray (Superbiochips, Seoul, Korea). Immunohistochemistry was performed in FFPE samples of the patient and tissue microarray of controls for OATP1C1 (1:200 dilution; Abcam Cat # ab234729, RRID: none), OATP2B1(1:200 dilution; Novus Cat # NBP1-80979, RRID : AB_11038565), MCT8 (1:500 dilution; Novus Cat # NBP1-89196, RRID: none), MCT10 (1:50 dilution; Novus Cat # NBP1-80700, RRID : AB_11011163), LAT1 (1:50 dilution; Abcam Cat # ab208776, RRID: none), and LAT2 (1:50 dilution; Novus Cat # NBP1-47989, RRID: none).

**Figure 3:**
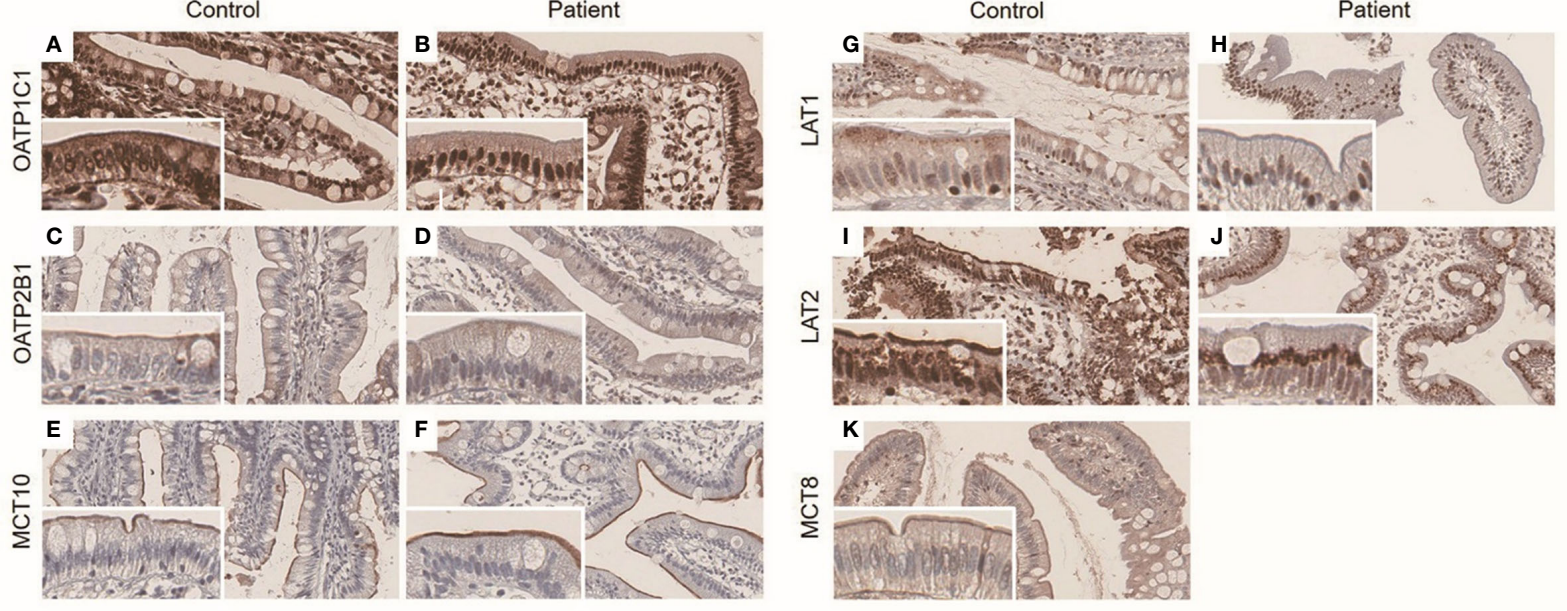
**(A–K)** Immunohistochemistry result of ileal LT4 transporters in distal ileum. The panels were taken at × 20 magnification, and inset represents × 40 magnification. OATP1C1 was expressed in both nucleus and brush border in control **(A)** and the patient **(B)**. OATP2B1 and MCT10 were mainly expressed in brush border in control **(C, E)** and the patient **(D, F)**, respectively. LAT1 was mainly expressed in brush border in control **(G)**, whereas there was perinuclear staining in the patient **(H)**. LAT2 was detected at both perinuclear area and brush border in control **(I)**, but was stained at only perinuclear area in the patient **(J)**. The expression of MCT8 was not able to check in the patient due to lack of remnant ileal tissue. It was mainly expressed in brush border in control **(K)**.

In terminal ileum, the staining patterns of OATP1C1, OATP2B1, and MCT10 were similar between control and the patient (**Figures 3A–F**). LAT1 and LAT2 were stained at brush border in controls, but both were not stained at brush border in the case patient - the staining was limited to nucleus or perinuclear area (**Figures 3G–J**). MCT8 was mainly expressed at brush border in controls (**Figures 3K**). Due to the lack of remnant tissue, the expression of MCT8 in terminal ileum could not be checked in the patient.

**Figure 4:**
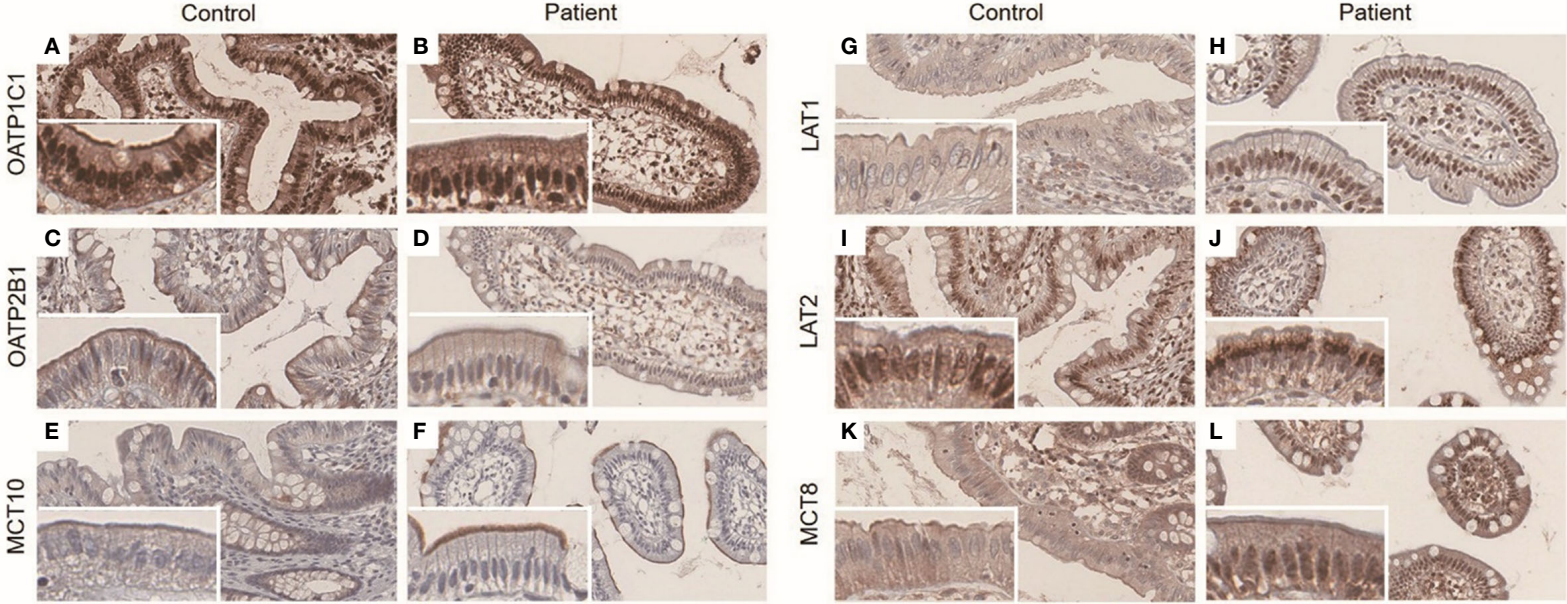
**(A–L)** Immunohistochemistry result of ileal LT4 transporters in ascending colon. The panels were taken at × 20 magnification, and inset represents × 40 magnification. OATP1C1 was expressed in both nucleus and brush border in control **(A)** and the patient **(B)**. OATP2B1 was expressed in brush border in control **(C)** and the patient **(D)**. MCT10 staining was weak and limited to brush border in control **(E)** and the patient **(F)**. LAT1 was mainly stained at brush border in control **(G)**, but was mainly stained at nucleus in the patient **(H)**. While LAT2 was stained at both nucleus and brush border in control **(I)**, it was stained at only perinuclear area in the patient **(J)**. MCT8 was expressed in brush border in control **(K)**, but was expressed in both nucleus and brush border in the patient **(L)**.

As LT4 was known to be absorbed mainly in jejunum and ileum (28), the functional importance of colonic transporters is unclear. However, because colonic FFPE sample of the patient was available, we also investigated the expressions of transporters in colonic epithelium to know the expression pattern was comparable with ileum, and found similar expression patterns. The staining patterns of OATP1C1, OATP2B1, and MCT10 were similar in both groups, as was terminal ileum (**Figures 4A–F**). Like terminal ileum, LAT1 and LAT2 were stained at brush border of control and LAT2 was also stained at nucleus. In the patient, the membranous expression of LAT1 and LAT2 was decreased as in terminal ileum (**Figures 4G–J**). MCT8 was expressed in brush border in control, which was similar to ileum, but was expressed in both nucleus and brush border in the patient (**Figures 4K–L**).

**Figure 5:**
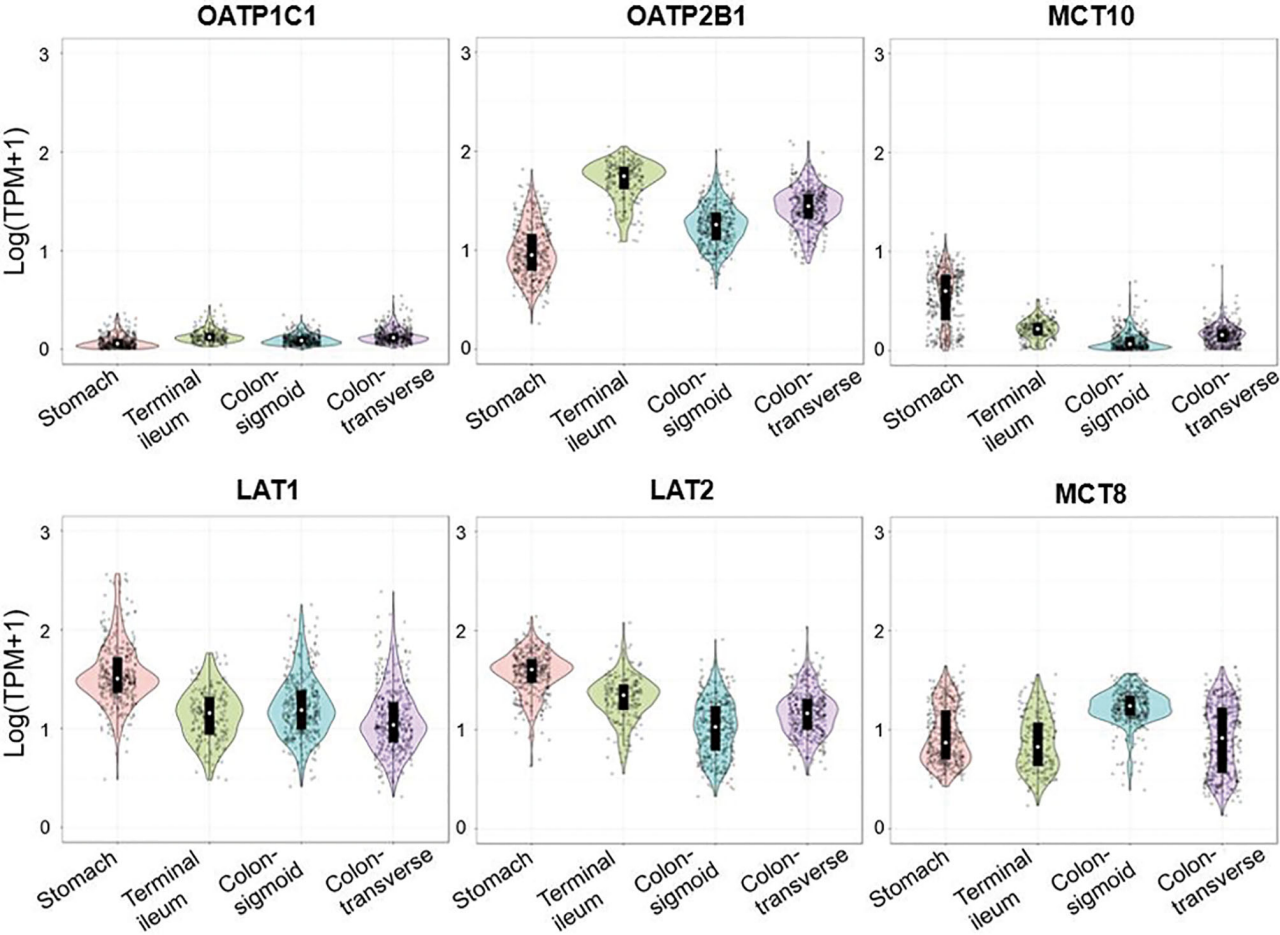
Expression of SLCO1C1 (OATP1C1), SLCO2B1 (OATP2B1), SLC16A10 (MCT10), SLC7A5 (LAT1), SLC7A8 (LAT2), and SLC16A2 (MCT8) in gastrointestinal tract. Violin plots were used to express the TPM data of transporters in each tissue. The colors indicate the different tissues. TPM, transcripts per million.

In order to check whether the expression of thyroid hormone transporters in ileum and colon is parallel, the expression pattern of LT4 transporters in large-scale healthy population was confirmed *via* Genotype-Tissue Expression (GTEx) data (**Figure 5**). We downloaded gene transcript per million (TPM) values from GTEx data consortium v8 data (dbGaP Accession phs000424.v8.p2) and each sample was annotated based on the sample information provided by GTEx portal. For data visualization, we used 359 stomach, 187 terminal ileum, 373 sigmoid colon and 406 transverse colon samples and TPM values were log-transformed. All plots were generated with the ‘ggplot2’ package implemented in R, and the expression pattern was similar in stomach, terminal ileum, transverse colon, and sigmoid colon in each transporter.

Currently, she is maintaining her euthyroid status by taking 9.1 - 10.0 μg/kg/day of LT4 depending on her body weight – 800 μg/day for 80 kg (10 μg/kg/day), 700μg/day for 76.9 kg (9.1 μg/kg/day), 600 μg/day for 64 kg (9.4 μg/kg/day) – which is about 6 times higher than usual dose of 1.6 μg/kg/day (5).

## Discussion

In this study, we found decreased membranous expression of LAT1 and LAT2 in epithelial cells of distal ileum and ascending colon, and aberrant expression of MCT8 in colonic epithelial cells in a hypothyroid patient who required 10 μg/kg of LT4 to maintain euthyroid status without any organic disorder.

When persistent hypothyroidism despite oral LT4 therapy is encountered, the first step is to check possible causes including dietary or medicinal interference, gastrointestinal malabsorption, nephrotic syndrome, hepatic disease, cystic fibrosis, and pregnancy (7, 8, 29). After excluding the above causes, it is necessary to identify pseudomalabsorption, namely nonadherence to LT4 therapy. According to the literature, most cases suspecting LT4 malabsorption were pseudo-malabsorption (4, 12). LT4 absorption test is a useful tool to diagnose pseudomalabsorption, but the protocol of the test and description of malabsorption has not been established in one way. Since the first analysis of LT4 absorption using oral ^125^I-thyroxine and intravenous ^131^I-thyroxine (30), Greenstadt et al. (31) established a nonisotope method using serum total T4 and volume of distribution [LT4 absorption rate [%] = peak Δtotal T4 (µg/dL) × Vd (dL) ÷ administered dose of LT4 (μg)] × 100, Vd = volume of distribution, 0.442 x BMI (32)]. Although there are different methods of performing the LT4 absorption test in aspects of LT4 dose (from 600 mg to 2,000 mg), the interval of administration of LT4 (daily intake or weekly intake), and description of pseudomalabsorption [TSH decrease by more than 40% from baseline after 2 hour (14), or whether fT4 increase by 50% of the initial value after 2 hour (33)], the Greenstadt’s formula was commonly used in Mayo clinic (34) and study of Sun et al. (13), and normal range of LT4 absorption was assumed 60-158%.

In the patient who required (800 μg/80 kg) 10 μg/kg of LT4 to maintain euthyroid status, we found the absorption rate of LT4 was 8.4% excluding pseudo-malabsorption. Because we could not find any organic causes of the LT4 malabsorption, we tried to investigate the expression of ileal transporters of the patient. Although most ileal absorption of oral LT4 occurs at jejunum and ileum (28), it was impossible to obtain specimens from those areas in the case patient because endoscopic biopsy is limited to stomach, duodenum, and colon. Therefore, we checked the expression of each transporter in various organs and found out that the expression pattern was similar in the small intestine and colon. On the premise that the transporters were expressed similarly in the small and large intestine, we compared the expression pattern in the terminal ileum and ascending colon between the patient and controls.

Interestingly, the membranous expression of LAT1 and LAT2 in epithelial cells of distal ileum and ascending colon in the patient was decreased. LAT1 and LAT2 have been mentioned in the *in vitro* study as candidate transporters responsible for T4 uptake in the intestine. In Caco2 model, the influx of T4 was inhibited by L-type amino acid transporter ligands including leucin and 2-aminobicyclo-(2,2,1)-heptane-2-carboxylic acid (BCH) (35). However, it is uncertain whether those results in Caco2 cells perfectly correspond to the real thyroid hormone transport in human enterocytes. In one study, LAT2 was reported to transport T3, but not T4 in *Xenopus laevis* oocyte (36). If this is the same in human ileal epithelial cells, the decreased ileal expression of LAT2 observed in the patient would be only an incidental finding. However, *Xenopus laevis* might differ from those human gut cells, and there remains a possibility of redundant function of each transporter according to the species, type, or location of cells. In another study with *Xenopus laevis* oocyte, both T3 and T4 influx by human LAT1 was reported (37), which may support our findings. It might be carefully supposed that the decreased membranous expression could be related with the malabsorption of LT4, and if LAT1 or LAT2 has a true function in human intestinal LT4 absorption, this result could be an important piece of human evidence.

In addition to the decreased membranous expression, we found an increase in perinuclear localization of LAT2 in ileal cells of the patient. There is endoplasmic reticulum in the perinuclear area, and it participates in the protein targeting pathway. When a preprotein is synthesized from ribosome, it moves into the endoplasmic reticulum by a signal sequence which is located in the amino-terminal of a polypeptide chain. This signal sequence is cleaved by signal peptidase and protein in turn travels through the Golgi complex and secretory vesicles to the target site (38). Considering this protein targeting process, the perinuclear staining of LAT2 would suggest excessive synthesis or dysfunction of signal peptidase causing decreased membranous expression of LAT2.

There also existed a difference in colonic expression of MCT8 between controls and the patient; it was limited to the membrane of epithelial cells in controls, whereas in the patient, it was expressed at both the nucleus and the membrane. The MCT8 staining was performed at the last part of the FFPE sample of the patient, thus, we could not compare its expression at the distal ileum due to no remaining specimen of terminal ileum of the patient. MCT8, a critical thyroid hormone transporter in the brain, has been known to have trivial expression and function in the intestine (39). There is a limitation in that the expression in the distal ileum could not be confirmed, but if it is similar to the expression in the colon, there is a possibility that MCT8 may contribute to intestinal transport.

Regarding the other transporters, the expression of OATP1C1, OATP2B1, and MCT10 was similar in both the patient and control group. Due to low expression of the OATP family in the human intestine, those transporters were expected to have little function in LT4 uptake in intestine, and the staining results were consistent (40). Another study using human colorectal adenocarcinoma cell line Caco2, which has been commonly used to mimic human intestinal epithelial cell (41, 42), reported that the influx of T3 by Caco2 cell was interfered by tryptophan and verapamil which are ligands of T-type amino acid transporter, for example, MCT10 (35). However, in our case, since synthetic T4 was mainly administered to the patient, MCT10 is thought to have contributed little to absorption disorder in our study.

The limitation of our study is that the decreased expression of LAT1 and LAT2 could be due to chronic hypothyroidism. However, the patient’s high TSH was detected at the beginning of the diagnosis, and the possibility of effect from hypothyroidism itself is low. Also, we could not evaluate the expression of transporters at jejunum and upper ileum, in which most gastrointestinal absorption of LT4 occurs, because the biopsy at these sites is impossible without surgery. Instead, we conducted our study using terminal ileum and ascending colon based on the GTEx data that the expression of transporters in the small and large intestine is similar. However, the findings of distal ileum could not represent exactly the changes of the upper small intestine. In addition, we could not evaluate the intestinal expression in hypothyroid patients taking LT4 without malabsorption. Moreover, we could not elucidate the direct relationship between LT4 absorption and transporter LAT1 and LAT2 in molecular levels.

However, this study would contribute to the understanding of the uptake of LT4 in the human intestine. This study is, as far as we know, the first human analysis of ileal LT4 transporter expression in a hypothyroidism patient. The other strength of our study is that the true malabsorption was distinguished from pseudomalabsorption in several ways including laboratory tests, endoscopic biopsy, imaging study, and an LT4 absorption test.

## Conclusion

The expression of thyroid hormone transporters in ileum has not been clarified, and should be considered as one of the causes of true malabsorption of LT4 in a refractory hypothyroidism patient who excluded interfering food or medication, poor compliance, and gastrointestinal absorption disorder. Our case showed a decreased membranous expression of LAT1 and LAT2 transporter in terminal ileum and ascending colon, and this result would provide information to investigate the role of intestinal transporters during the absorption of LT4 in the human intestine.

## Data Availability Statement

The original contributions presented in the study are included in the article. Further inquiries can be directed to the corresponding authors.

## Ethics Statement

The authors have obtained written informed consent from the case patient to share data and images. The consent for controls was waived according to the approved protocol by the Institutional Review Board of Seoul National University Hospital and based on the Declaration of Helsinki for Medical Research.

## Author Contributions

Concept and design: CWC and YJP. Acquisition of data, literature review, and refinement of manuscript: All authors. Analysis and interpretation of data: CWC, JMB and YJP. Manuscript writing: CWC. Review of final manuscript: YJP. All authors contributed to the article and approved the submitted version.

## Conflict of Interest

The authors declare that the research was conducted in the absence of any commercial or financial relationships that could be construed as a potential conflict of interest.
